# Supplementary material for: A mixed-method study of feasibility and acceptability of the Dapivirine vaginal ring among adolescent girls and young women (AGYW) in selected Zimbabwean districts
Source: PLOS Glob Public Health. 2025 Mar 31;5(3):e0004081. doi: 10.1371/journal.pgph.0004081 (PMC11957275; doi:10.1371/journal.pgph.0004081)
Supplement: Attachment — Submitted filename: responses to reviewers DPV ring manuscript.docx [file pgph.0004081.s003.docx]

| **Comment** | **Response** |
| --- | --- |
| 1. Methodology: please explain where the AGYW were recruited from. Did you recruit women attending health facilities? Did you partner with local organizations? The recruitment strategy is not explained in the paper and yet is essential for the interpretation of your results. | The initial sampling frame consisted of AGYW accessing HIV and or sexual and reproductive health services public sector health facilities and Population Solutions for Health funded health care centres (New Start Centres) in the districts presented in table 3. The recruitment strategy was a random sample of participants voluntarily consenting to receive either oral or DPV-VR as their preferred PrEP method. |
| 2. Table 2 is confusing. Your 'Total PrEP' column adds up to 1,596, which is the grand total of study participants; however, only 390 participants opted for PrEP. Please clarify. Furthermore, shouldn't your DPV acceptability vs PREP acceptability by location be based on the total N for each location? For instance, what proportion of participants from Bulawayo opted for DPV vs PrEP, in which case your total N would be the total number of participants from Bulawayo and so on for the other locations? Anyway, please review Table 2 and explain your decisions or the math that informs your analysis in a footnote for the reader. | 390 accepted oral PrEP while 1206 accepted DPV-VR giving a total of 1596. We have added a row with totals after Age variable to make this clear. Also note comment made with reference to PrEP should be “oral PrEP” to make it clear since DPV-VR is also PrEP.  The primary purpose was to demonstrate preference between Oral PrEP and DVR-VR hence the denominator is total accepting either of the 2 PrEP options. The study only recorded this denominator as these were the actual recruited in the study (1596). So the actual total number of recorded participants in Bulawayo for example is 364 |
| 3. Qualitative results: One key strength of your paper is your mixed-method design. Could you provide some participant excerpts to support the narrative that you provided. This will help augment the value of qualitative research in your study. In the qualitative section, you refer to participants as 'recipients of care' but do not use this term when reporting your quantitative data. I suggest that you simply use the term 'participants' unless you explain how your participants are 'recipients of care', that is, what is the care that they are receiving outside the context of the study. | 3 excerpts added in the qualitative section. The term recipients of care is replaced by participants |
| 4. You report that the HIV incidence among DPV users and PrEP users was 'comparable': it is not clear how you are using the word 'comparable'. Additionally, the HIV incidence among DPV users is THREE TIMES that of PrEP users (2.32 per 100 person years vs 0.67 per 100 person years). This is a hardly 'comparable'; please revise or explain what you mean. Did you carry out any statistical tests to assess if this difference is statistically significant? If you did, please include the p-value for this. | While the incidence point estimates seemed different, the statistical test did not show any significant differences as evidenced by the overlapping 95% confidence intervals at 5% significance level presented. A p-value of 0.6080 has been added on the revised version. This is a limitation coming from the oral PrEP results with only 2 sero-conversions out of the 390. This means the CIs retained will be wide due to resulting number of person years. The limitation has been added in the manuscript. |
| 5. Discussion: this section needs to be strengthened. Tell the reader what the implications of your findings are: what do we learn about the factors that contribute to DPV acceptability? | The discussion section has been strengthened to respond to the reviewers’ suggestions |
| 6. Please do a thorough copyedit of the paper. There were several small grammatical errors that distracted from an otherwise engaging paper. | The document has been reviewed and several grammatical errors were fixed |
| 1. The authors failed to recognize that religion is a key variable in health-seeking behaviour and adherence in Zimbabwe, especially for individuals belonging to certain apostolic groups. The authors need to report on the study population's religious group variable and use this lens in their analysis, discussions and recommendations. | Religion added in both results and discussion sections |
| 2. We also want to know the alcohol and drug use patterns of the sample and this needs to be included in the analysis. | Drug use and alcohol questions not asked in the study |
| 3. The authors failed to outline clearly the limitations of their study, this is a key weakness of the manuscript | Study limitations added at the end of the discussion section. |
| 4. The title ‘A mixed-method study of feasibility and acceptability of the Dapivirine vaginal ring among adolescent girls and young women (AGYW) in Zimbabwe’ is misleading as it implies that a statistically representative sample covered the whole country. The title needs to be changed to something like ‘A mixed-method study of feasibility and acceptability of the Dapivirine vaginal ring among adolescent girls and young women (AGYW) in selected study site in Zimbabwe | Title updated to reflect comment |
| 5. The manuscript fails to make any detailed recommendations to policymakers in Zimbabwe and other countries in southern Africa other than stating the obvious. | Recommendations and conclusions added |
